# Long-Chain 3-Hydroxyacyl-CoA Dehydrogenase Deficiency (LCHADD)-Associated Ocular Pathology—A Narrative Review

**DOI:** 10.3390/diagnostics16020295

**Published:** 2026-01-16

**Authors:** Magdalena Hubert, Maciej Gawęcki

**Affiliations:** 1 Department of Ophthalmology, Pomeranian Hospitals, 84-200 Wejherowo, Poland; magdalenahubert111@gmail.com; 2 Dobry Wzrok Ophthalmological Center, 80-392 Gdansk, Poland

**Keywords:** long-chain 3-hydroxyacyl-CoA dehydrogenase deficiency, chorioretinopathy, macular neovascularization, myopia

## Abstract

Long-chain 3-hydroxyacyl-CoA dehydrogenase deficiency (LCHADD) is an extremely rare autosomal recessive disorder, with only a few hundred affected individuals worldwide. Since its initial recognition in the 1980s, only a limited number of studies have described its ocular manifestations. The aim of this review was to summarize and organize the available published evidence regarding ocular findings in LCHADD and their classification. A PubMed search was conducted for studies describing ocular findings associated with LCHADD, using combinations of the following keywords: LCHADD, chorioretinopathy, ocular findings, vision, therapy, and long-chain 3-hydroxyacyl-CoA dehydrogenase deficiency. The review included studies published within the past 20 years that reported at least six cases. The search identified 11 eligible studies. Findings were grouped into three categories: LCHADD-associated chorioretinopathy, macular neovascularization (MNV), and the effects of dietary therapy on visual function. Chorioretinopathy emerged as the major pathognomonic ocular feature of LCHADD. MNV was reported in approximately 20% of eyes, often bilaterally but not simultaneously. Progressive myopia was observed in most patients. Newborn screening and early initiation of dietary therapy appear critical and may slow the progression of chorioretinopathy. A strong correlation between patient age and chorioretinopathy severity was consistently demonstrated, and visual deterioration occurred even in individuals with good metabolic control. LCHADD is a life- and vision-threatening disorder characterized by a distinctive chorioretinopathy present in nearly all patients. Early detection through newborn screening and regular ophthalmic follow-up is essential for the optimal management of affected individuals.

## 1. Introduction

Long-chain 3-Hydroxyacyl-CoA dehydrogenase (LCHAD) deficiency (LCHAD deficiency—LCHADD) (OMIM # 609016) is an extremely rare metabolic disorder affecting the mitochondrial beta-oxidation of long-chain fatty acids (LCFAO) [1,2]. The underlying reason for this pathology is a mutation in the *HADHA* gene encoding the alfa-subunit of the mitochondrial trifunction protein (TFP), impairing the activity of long-chain 3-Hydroxyacyl-CoA dehydrogenase [3]. The other enzymatic activity of TFP (long-chain enoyl-CoA hydratase, long-chain 3-hydroxyacyl-CoA dehydrogenase, and long-chain 3-ketoacyl-CoA thiolase) is preserved. The dysfunction in long-chain fatty acids dehydrogenase not only comprises the energy supply to the cell, but also causes the accumulation of partially oxidized long-chain 3-hydroxy fatty acids and its derives—3-hydroxyacylcarnitines (3-OH-AcS) [4,5,6]—that are believed to cause cytotoxicity to a range of susceptible cells [3,7,8].

Elevated 3-OH-AcS are a marker of the impaired activity of both LCHAD (LCHADD) and TFP (TFP deficiency—TFPD). Nowadays, in many countries the disease is diagnosed by newborn screening (NBS) with tandem mass spectrophotometry of a dried blood sample to estimate the levels of acylcarnitines [5,6]. Distinguishing LCHADD from TFPD requires the identification of isolated LCHADD on an enzymatic assay in lymphocytes or skin fibroblasts. TFPD is confirmed by the identification of deficiencies in all three TFP enzymatic activities: enoyl-CoA hydratase, long-chain 3-hydroxyacyl-CoA dehydrogenase, and long-chain 3-ketoacyl-CoA thiolase in lymphocytes or skin fibroblasts. Genetic testing confirms the mutation. However, no consistent clinical diagnostic criteria for LCHADD or TFPD have been acknowledged so far [9].

LCHADD is an autosomal recessive disease and a typical mutation c.1528G>C (rs137852769, p. Glu510Gln) substitution in exon 15 *HADHA* gene [10] is almost always detected in at least one alle [5]. Compound heterozygosity for the c.1528G>C and a second *HADHA* allele polymorphism may result either in isolated LCHAD deficiency or in general TFP deficiency. The prevalence of c.1528G>C mutation is distinctly high in the Pomerania district in northern Poland, where carrier frequency is 1:73 and morbidity is 1:16,900, which gives the highest ever noted [5]. The reason for this is a founder effect—Nedoszytko et al. published a study [10] where the frequency of carriers of the c.1528G>C (rs137852769, p. Glu510Gln) mutation among patients of Kashubia origin was 1:57. (Kashubia is a region and an ethnic group in the Pomerania region in Poland that has been noted in historical documents since the thirteenth century. They live in a closed community protecting their language and culture, and therefore an endogamy is not uncommon.) Other *HADHA* variants have also been reported [10,11]. The disease is generally more frequent in countries surrounding the Baltic Sea—Finland, Sweden, Estonia, the Netherlands, and Poland—while hardly detectable in Asia [2,12,13]

LCHADD typically presents with hypoketotic hypoglycemia, rhabdomyolysis, and liver dysfunction that occur especially after fasting or during illness [13] and exertion. Moreover, cardiomyopathy as a result of the accumulation of by-products of defected fatty acid oxidation (FAO) may result in cardiac arrest. Neuropathy is also an additional finding. Chorioretinopathy is pathognomonic for LCHADD [14] and together with other ocular abnormalities appears to be one of the major burdens for affected individuals, significantly impacting the quality of life of both patients and their families [15].

The therapy consists of a dietary regimen limiting total long-chain fatty acid (LCFA) intake to 10%, combined with 10% medium-chain triglyceride (MCT) supplementation [16,17]. Avoiding fasting, infections, and exertion is crucial. The administration of carbohydrates during increased metabolic stress is also required [18].

With the recommended therapy, most patients survive into childhood, and the longest-living individuals reach their 30s [6,15]; however, acute hypoketotic hypoglycemia remains a major cause of mortality [13,19].

## 2. Material and Methods

The PubMed database was searched using the following terms: LCHADD, chorioretinopathy, ocular findings, vision, therapy, and long-chain 3-hydroxyacyl-CoA dehydrogenase deficiency. The search results were divided into three groups. The first group included studies describing LCHADD-related chorioretinopathy and other ocular manifestations (Table 1). The second group included studies on macular neovascularization associated with LCHADD (Table 2). The third group comprised studies addressing dietary therapy and its potential visual outcomes (Table 3). Only studies published within the past 20 years and including at least six patients were considered eligible for review. Individual case reports and papers lacking precise diagnostic data were excluded from this review. This exclusion criterion was applied, first, to facilitate a more robust statistical evaluation of the incidence and clinical follow-up of specific symptoms. Second, the past 20 years have seen the advent of modern imaging modalities, which have significantly enhanced the sensitivity for detecting ocular pathologies within the ocular fundus.

Due to the rarity of the disease and the limited number of available studies on LCHADD and its ocular manifestations, the search period was extended to 25 years for analyses focused on the effects of dietary treatment on LCHADD-related chorioretinopathy. Some studies that did not fully meet the inclusion criteria were referenced only when data regarding a specific issue were scarce or unavailable.

## 3. Results

The search revealed 11 studies that met the criteria (Table 1, Table 2 and Table 3). Four of them [6,16,23,25] were based on the same group of 40 patients—37 with LCHADD and 3 with TFPD—described together as a one group (two of these studies described chorioretinopathy (Table 1) [6,23], one choroidal neovascularisation (Table 2) [25], and one described the effect of dietary therapy on visual outcomes [16]). Although the four analyzed studies were based on the patients from the same LCHADD and TFPD chorioretinopathy natural history study, their objectives were different, and thus different data could have been extracted. Another two studies were based partially on the same group [12,20]—the first study over time was expanded with data on two additional subjects. Finally, the remaining two studies [24,26] were based on the same group, yet they considered different issues. Therefore, in this review one was presented with the studies regarding chorioretinopathy (Table 1) [24], while the other with studies referring to LCHADD and MNV comorbidly (Table 2) [26].

The ocular manifestation that is pathognomonic for the LCHADD is chorioretinopathy [14,27]. Other ocular findings that have been reported include macular neovascularization or submacular fibrosis, as well as progressive myopia. Additional observations include staphyloma, epiretinal membrane, and macular edema.

### 3.1. Chorioretinopathy

Chorioretinopathy associated with LCHADD was first mentioned in 1992 by Bertini et al. [28] in a case report. It was well described in 1997 by Tyni et al. [29] and the first staging was proposed in that study.

Patients with LCHADD typically have a normal fundus appearance at birth (Tyni stage 1). With time—usually by the age of two, or in those who survive long enough—pigmentary retinopathy develops in the posterior pole (stage 2). In subsequent stages, circumscribed and progressive chorioretinal atrophy emerges, initially sparing the fovea (stage 3) and later involving the entire posterior pole (stage 4). The staging of LCHADD chorioretinopathy was refined by the same author [30] in 2012. Stage 2 was subdivided into substages according to the intensity of pigmentary deposits (P1–P3) and the severity of atrophy (A1–A3) and reference pictures were given.

In 2024, Wongchaisuwat at el. gave a detailed description of the LCHADD chorioretinopathy and typical findings in optical coherence tomography (OCT), widefield fundus autofluorescence (FAF), and full-field electroretinography (ffERG) and provided new staging based on multimodal imaging results [23]. It was derived from original staging by Tyni [19] but provided important modifications. Stage 1 refers to a fundus of a child without chorioretinopathy, as well as a lack of alterations in ERG, widefield FAF, or in OCT. Stage 2 is subdivided into 2A and 2B and presents pigmentary deposits that surpass (2B) or do not (2A) the vascular arcades. The damage to the retinal pigment epithelium (RPE) is reflected in the speckled foci of hypo/hyperautofluorescence on widefield FAF. The attenuation of the ellipsoid zone (EZ) and the RPE, along with normal to thickened RPE–Bruch complex in macula (2A) or fovea (2B), are observed on OCT. Full-field ERG is mildly to moderately decreased. While chorioretinal atrophy becomes visible and pigmentary deposits reach the periphery in stage 3, ffERG response decreases and well-demarked hypofluorescence lesions resulting from RPE loss are observed on FAF. Optical coherence tomography demonstrates the progression of the degenerative process: attenuation of the outer retina with outer retinal tubulations (ORTs) in stage 3A followed by retinal atrophy in 3B, along with choroidal changes—thinning (3A) and atrophy (3B). It is characteristic that chorioretinal atrophy starts at the peripapillary margin and within the paracentral macula and eventually spreads from vascular arcades to the peripheral fundus [23,31]. In stage 3, fovea is spared, probably due to the peninsulas of residual choroidal vasculature branching toward fovea, permitting the preservation of a central vision [31]. In the visual field, it is typically manifested by paracentral scotoma, which—with time—increases its density and spreads [21,22].

These lesions progressively increase in both severity and extent, ultimately involving the periphery and the fovea in stage 4. The progression is reflected by the enlargement of hypoautofluorescent lesions on FAF imaging, extensive choroidal and outer retinal atrophy on OCT, and functional decline on full-field electroretinography (ffERG), which in some cases may lead to an extinguished retinal response.

OCT findings indicate that LCHADD pathology affects the outer retinal layers first and most severely, with inner retinal involvement occurring secondarily. Early LCHADD-related chorioretinopathy is characterized by irregularity of the interdigitation zone, followed by loss of the ellipsoid zone and the underlying RPE, with abrupt transition zones between healthy tissue and affected areas. The margins of atrophic regions are often delineated by outer retinal tubulations (ORTs), a feature also observed in other degenerative retinopathies [21,32]. This suggests that the loss of the RPE precedes the loss of photoreceptors, and that the formation of ORTs may represent a compensatory strategy to preserve photoreceptors in the setting of diminished trophic support from a degenerating RPE and choriocapillaris [21].

The OCT angiography (OCTA) allowed us to assess the vascular changes—significant vessel density (VD) loss and increased nonperfusion areas (NPA) in the deep choroidal plexus (DCP) and choriocapillaris (CC) [33]. Marked choriocapillaris loss was substantially more extensive than photoreceptor loss and corresponded closely to areas of RPE loss detected on fundus autofluorescence [21].

Deterioration in the OCTA parameters correlated positively with the severity of the LCHADD systemic symptoms and negatively with BCVA. Interestingly, vessel density and areas of nonperfusion at choriocapillaris correlated negatively with the plasma level of acylcarnitine [21].

Although the pathomechanism of chorioretinopathy remains unclear, the sequence of the changes visualized in the OCT and OCTA provides important clues and several studies have proposed the underlying pathways.

Sannon J. Babcock et al. [8] created a mouse model of LCHADD-associated chorioretinopathy. They observed a 5- to 7-fold increase in acylcarnitine staining within the RPE and sclera of the LCHADD model compared with wild-type mice. The RPE in the model was disrupted, exhibiting loss of basal infoldings and apical microvilli, as well as impaired interdigitation with the outer photoreceptor segments, which were also abnormal. These findings are consistent with the study by Tyni et al. [3], which suggested that the RPE—rather than the choriocapillaris—may be the primary cell layer affected in LCHADD retinopathy. The immunohistochemistry of frozen ocular sections demonstrated that TFP is dominant in RPE energy production and is not present in other retinal layers. Furthermore, the LCHADD model showed the downregulation of differentially expressed genes in the RPE and sclera.

Padmini P. Polinati [7] derived patient-specific RPE monolayers from individuals with LCHADD and demonstrated their primary cellular pathology. The RPE cells were approximately 2.5 times smaller, exhibited irregular morphology, and showed disorganized tight junctions, which triggered apoptosis. They also displayed marked cytoplasmic accumulation of neutral lipids, reduced pigmentation, fewer melanosomes, and an increased number of melanolysosomes

Among the studies identified in our search, four examined the correlation between chorioretinopathy progression and the type of diagnosis. Two of these were conducted by the same author and included partially overlapping patient cohorts, with some additional subjects enrolled later. The studies compared patients diagnosed through newborn screening or family history with those diagnosed symptomatically—typically after episodes of hypoglycemia—which in most cases resulted in a substantially later diagnosis. One of the studies [17] found no correlation between the progression of chorioretinopathy in patients diagnosed presymptomatically and those diagnosed after symptom onset. In contrast, three studies demonstrated that chorioretinopathy was less pronounced in patients with an early diagnosis and early initiation of dietary therapy (those identified through newborn screening or family history), whereas it was more severe in children diagnosed later, after the onset of LCHADD-related symptoms [6,12,20]. The most likely explanation for this disparity is the accumulation of hydroxyacylcarnitines in vulnerable tissues during periods of hypoglycemia or physiological stress prior to diagnosis and the initiation of appropriate dietary treatment. The analyzed studies showed no clear correlation between current acylcarnitine levels, the number of metabolic crises, and the stage or progression of ocular disease [22] nor with ERG outcomes [12].

Our search revealed that most patients maintain good visual acuity (VA) in at least one eye [21,24]. Preservation of good vision in one eye may contribute to the late diagnosis of macular neovascularization (MNV)—often detected only at the stage of scarring [23,25] because young patients may not notice visual decline in the fellow eye [25,33]. The impaired VA, defined as less than 1.0 on the Snellen chart, was observed in 42–66% of eyes (Table 1). Boese et al. [21] reported a linear decline in VA, with a slope of approximately 0.034 logMAR units per year.

**Figure 1:**
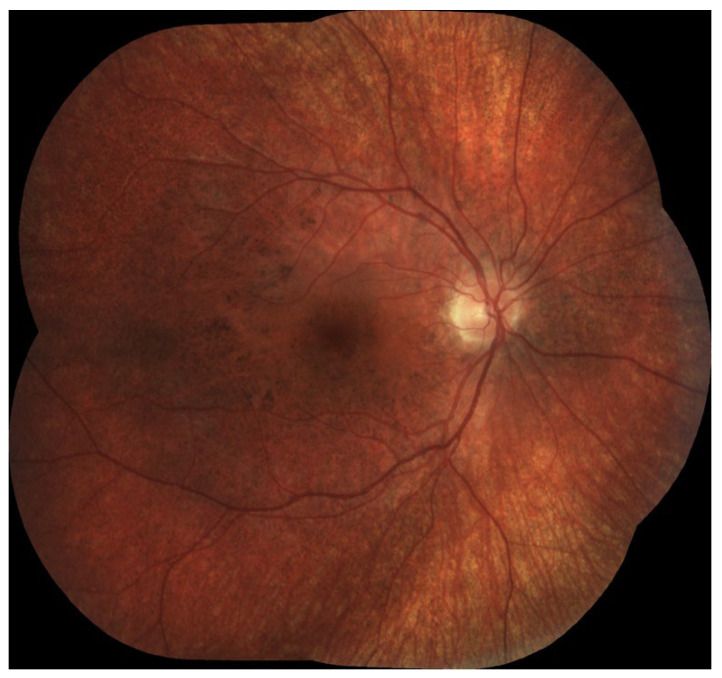
Chorioretinopathy stage 2. Visible atrophy of RPE with pigment accumulation in the macula, pigment clumping in peri-arcade areas, and salt-and-pepper look that spread to the mid-periphery.

**Figure 2:**
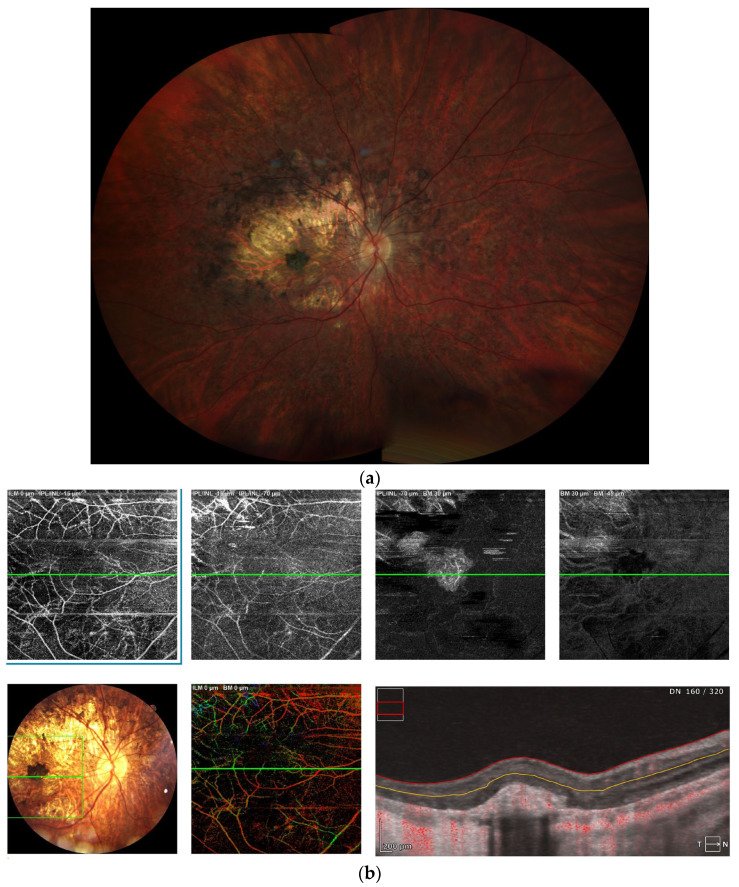

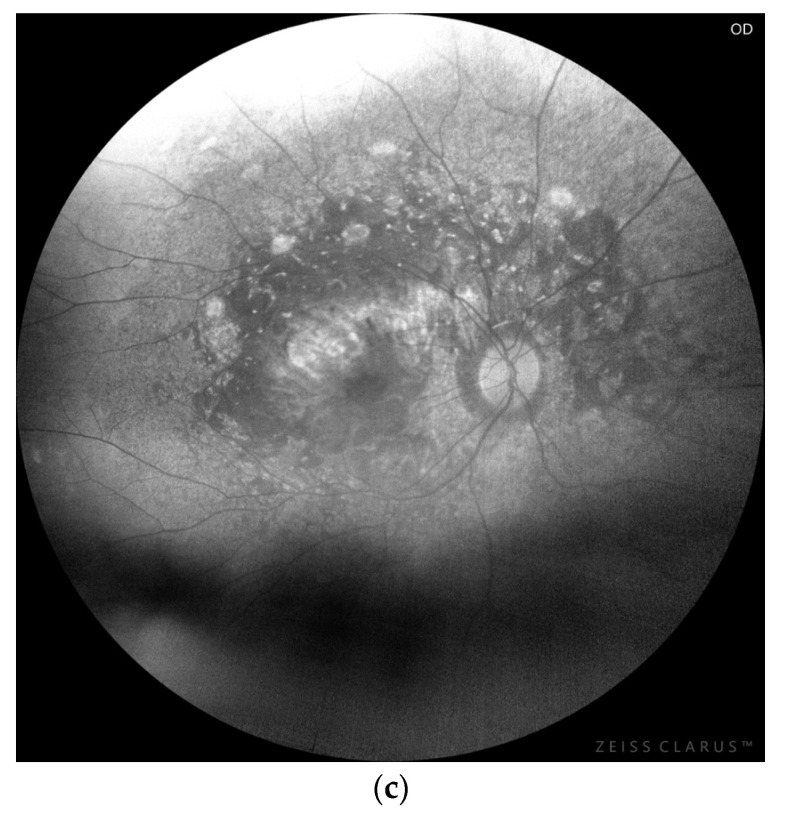
(**a**) Chorioretinopathy stage 3 with pigmented fibrotic scar in the fovea; advanced atrophy in the posterior pole, sparing the fovea; intense pigment clumping in the peri-arcade area; periphery is spared (**b**) OCTA—well formed CNV in deep plexus. (**c**) FAF presenting well-demarked hypo/hyperautofluorescence lesions from chorioretinal atrophy.

All analyzed studies demonstrated that chorioretinopathy was present in all LCHADD patients older than 3.6 years (Table 1), with some studies reporting detectable changes in all subjects from as early as 1 year of age, and others in all patients older than 2 years. The progression of chorioretinopathy was strongly correlated with age and was also associated with declining visual acuity. However, in cases complicated by macular neovascularization (MNV) or scarring, visual acuity was worse than expected based on the chorioretinopathy stage alone [23]. Examples of LCHADD chorioretinopathy are preseented in Figure 1 and Figure 2.

Among the studies identified in our search, those that included ERG as part of the ophthalmological evaluation confirmed that both rod and cone responses were impaired, corresponding to decreased night and color vision. ERG findings deteriorated progressively with increasing age and advancing disease stage.

### 3.2. Myopia

The search identified four studies that described myopia as an important symptom associated with LCHADD [12,20,21,24]. Myopia was reported in 30–100% of eyes (Table 1), with some cases demonstrating high myopia. Boese et al. observed a consistent progression pattern, with a mean decline in the spherical equivalent of −0.24 diopters per year, continuing into early adulthood—unlike typical juvenile myopia. The refractive error did not differ significantly between fellow eyes, in contrast to best corrected visual acuity (BCVA), which often showed asymmetry [21].

### 3.3. MNV

Two studies meeting the search criteria described macular neovascularization (MNV) in LCHADD (Table 2). MNV was reported in 21–25% of patients and in 19–27% of eyes, and was bilateral in 22–50% of affected individuals. OCTA revealed type 2 MNV in most cases [34]. In the majority of eyes, the MNV was inactive; nevertheless, it contributed to the deterioration of visual acuity. MNV associated with LCHADD was typically small, localized in the fovea, and prone to spontaneous regression or responsive to intravitreal anti-VEGF therapy. Although it shares several features with myopic MNV, in LCHADD it can also develop in eyes with emmetropia or only mild myopia. The pathogenesis of LCHADD-associated MNV remains unclear. A possible mechanism may overlap with that of myopic MNV: chorioretinal atrophy and substantial loss of choroidal tissue may lead to hypoxia and the subsequent upregulation of angiogenic factors. Additionally, the formation of cracks in Bruch’s membrane may trigger inflammatory responses and further stimulate the expression of growth factors [35,36].

### 3.4. Other Ocular Findings

Staphyloma was found in 25–44% of eyes. One of the studies revealed that staphyloma was present in all eyes in stage 4, and in none of the eyes in stages 1–2 [23].

Cystoid macular edema (CME) was observed in 26% of eyes [23] and epiretinal membrane (ERM) in 15% of eyes [23].

### 3.5. Treatment

The search identified only one study published within the past 25 years that examined the correlation between dietary treatment and visual outcomes (Table 3) [16]. It also revealed one review on this subject [37] but its content did not meet the criteria adopted for the review.

Dietary recommendations for patients with LCHADD include strict avoidance of fasting: the maximum fasting period is 3 h for children aged 0–3 years, 4–5 h for preschool and primary school children, and no more than 6 h for adolescents and adults. Intake of long-chain fatty acids (LCFA) should be limited to 10% of total energy intake (with 3–4% provided as essential fatty acids), while medium-chain fatty acids should supply 10–20% of total energy. The diet should also be supplemented with vegetable oils, included within the 10% LCFA allowance, to ensure adequate essential fatty acid intake. Protein and carbohydrate intake should be adjusted to the patient’s age and body weight, with an emphasis on complex carbohydrates such as cornstarch. A daily multivitamin and mineral supplement containing all fat-soluble vitamins should also be provided [17,18].

One study identified by our search [16] aimed to determine whether docosahexaenoic acid (DHA) supplementation could prevent the progression of LCHADD-associated chorioretinopathy, given that DHA deficiency is linked to severe, progressive pigmentary retinopathy. DHA cannot be synthesized from short-chain fatty acids in humans and must be obtained through the diet, for example, from fish rich in essential fatty acids. It can also be synthesized from its 18-carbon precursor, α-linolenic acid; however, this metabolic pathway is impaired in LCHADD. Gillingham et al. [16] demonstrated that visual acuity, measured by visual evoked potentials (VEPs), improved over time with DHA supplementation, regardless of baseline DHA status (normal or low). There was also a trend toward a positive correlation between VEP responses and plasma DHA concentrations. Long-chain 3-hydroxyacylcarnitine levels were negatively correlated with maximum ERG amplitude; however, no differences in VEP results were observed between subjects with low and high cumulative hydroxyacylcarnitine exposure. Neither plasma DHA concentration nor age independently predicted ERG outcomes. Optimal dietary therapy [38]—reflected by low plasma 3-hydroxyacylcarnitine and high plasma DHA concentrations—was associated with the preservation of retinal function and VA (as measured by VEP) [16]. Unfortunately, reliable DHA supplements with minimal contamination by other long-chain fatty acids remain scarce. Moreover, a recently published study reported no correlation between current dietary regimen and acylcarnitine levels [4].

There has been an ongoing debate regarding the use of triheptanoin (C7—an odd-chain triglyceride that appears more effective than MCT oil in reducing acute events such as hypoglycemia and rhabdomyolysis and in improving cardiac function) as an alternative to standard medium-chain triglyceride (MCT) oil therapy [18,39]. Other studies examining specialized infant formulas containing varied proportions of MCT and essential fatty acids [40], as well as methods for the precise calculation of energy equivalents [41] to achieve strict dietary control, also appear promising.

In cases of metabolic decompensation, intravenous administration of glucose and/or dextrose is recommended. The impact of metabolic decompensation on the progression of chorioretinopathy or the development of MNV remains unclear; some authors have suggested a positive correlation, whereas others, using acylcarnitine levels as a presumed cumulative marker of dietary adherence, found no such association [16,22].

## 4. Discussion

LCHADD is an extremely rare condition, first described less than 40 years ago, and the oldest known survivors may only reach approximately 40 years of age. As a result, the full spectrum of clinical manifestations has not yet been completely characterized. The largest published cohorts include around 40 patients. Many studies evaluate LCHADD and TFPD together, despite clear differences between these entities, which may significantly influence the reported statistics [6,18,21,23,25].

Ocular manifestations of the disease evolve with patient age. Because most affected individuals are children, many ophthalmic examinations are difficult to obtain or require general anesthesia, and thus should not be performed without a strong indication. Even the most recent staging system for LCHADD chorioretinopathy, proposed by Wongchaisuwat in 2024, relies on examinations that must be performed under general anesthesia in young infants [23]. Furthermore, this staging system may still be incomplete, as the role of macular neovascularization may be underestimated.

Although ocular manifestations are strongly age-dependent, each study includes a small number of patients across a wide age range. Because of this heterogeneity and the rarity of the condition, it is challenging to perform reliable statistical analyses or correlate disease progression with age and other clinical factors. Our search identified only a few studies on ocular findings published after 2000. Several limitations apply: most studies were retrospective, with follow-up durations ranging from months to years; participant ages varied greatly (from a few months to 36 years), even within the same study; diagnostic methods have evolved considerably over time, making direct comparisons between patients examined decades apart unreliable; and dietary recommendations have changed, with an ongoing lack of consensus regarding supplementation with DHA and related nutrients [18,37,38,39,40,41].

## 5. Limitations

LCHADD is an ultra-rare metabolic disorder, which inherently limits the volume of the available literature. The existing reports are frequently based on relatively small patient cohorts, a factor that often precludes robust statistical analysis regarding the prevalence and incidence of specific clinical symptoms.

Furthermore, diagnostic modalities for detecting retinal changes have advanced significantly over the last two decades. The sensitivity for identifying retinal lesions, such as MNV, has increased substantially with the introduction of high-resolution imaging. Consequently, earlier reports may have underestimated the true occurrence of these pathologies.

Similar constraints apply to the longitudinal monitoring of LCHADD chorioretinopathy; the precision offered by modern FAF was not available in older studies. Therefore, the collective data on LCHADD—particularly regarding the numerical prevalence and statistical trends—must be interpreted with caution.

## 6. Conclusions

Despite its limitations, this review provides a comprehensive overview of the current knowledge regarding ocular pathology in LCHADD. LCHADD is a potentially fatal condition, and its ocular findings remain a hallmark of disease recognition and progression. Newborn screening, early initiation of dietary therapy, and consistent treatment adherence are essential for patient survival. Individuals with LCHADD require close ophthalmic monitoring to enable the early detection of treatable complications, such as macular neovascularization. Longer-term follow-up of larger, more homogeneous patient cohorts, together with clinical trials aimed at establishing standardized guidelines for dietary management and MNV treatment, will be crucial for improving future care recommendations for patients with LCHADD.

## Figures and Tables

**Table 1:** Studies analyzing onset and evolution of chorioretinopathy in LCHADD patients published in recent 20 years.

Study	Study Design	No of Patients	Population	Results
Kristina T. Fahnehjelm et al., 2008 [20]	• retrospective case series • median follow-up 7.5 years (range 2.3–14.8 years) • clinical examination, ERG	10 patients	• patients diagnosed with LCHADD from two hospitals in Sweden	• chorioretinopathy first noticed at the age of median 3.6 years (range: 14 months–6 years) • chorioretinopathy present in both eyes in all patients • chorioretinopathy was less pronounced in patients with earlier diagnosis and prompt initiation of dietary therapy • myopia in 6/20 eyes (including—4/6 high myopia) • BCVA impaired in 12/18 eyes (no data regarding BCVA from one patient) • ERG pathological or subnormal in 14/20, extinguished in 0/20 • epiretinal gliosis in 1/20 eyes • staphylomas in 2 patients • fibrotic scar in 1/20 eyes • photophobia in 7/9 patients (no data regarding photophobia from one patient) • nystagmus in 1/10 patients
Kristina T. Fahnehjelm et al., 2016 [12]	• retrospective case series • dicarboxylic acids and/or acylcarnitine assessment	12 patients	• patients diagnosed with LCHADD from two hospitals in Sweden (12/16 diagnosed with LCHADD living in Sweden at that time)	• chorioretinopathy diagnosed in 22/24 eyes (the only patient who did not present with chorioretinopathy was 1 year old) • myopia in 10/24 eyes • BCVA impaired in 10/20 eyes; 4/20 eyes considered blind according to WHO definition • fibrotic scar in 1/24 eyes • ERG pathological or subnormal in 20/24 eyes, observed deterioration over time, not correlated to BCVA and acylcarnitine levels • staging
Erin A. Boese et al., 2016 [21]	• retrospective case series • follow-up: median 5.6 years (range 0.3–20.2 years) • evaluation of plasma acylcarnitine levels	18 patients with LCHADD (including 3 with TFPD)	• all patients managed at the Oregon Health & Science University (OHSU) Casey Eye Institute between 20 September 1994 and 18 August 2015 with diagnosis of either LCHADD or TFPD	• chorioretinopathy present in all patients older than 2 years of age • progressive atrophy of the outer retina, often preceded by the formation of outer retinal tubulations and choriocapillaris dropout on OCT • progressive myopia in 26/34 eyes with mean decline in spherical equivalent of −0.24 diopters per year, continued into early adulthood • BCVA impaired in 19/36 eyes—linear decline with slope of 0.034 logMAR units per year, notable disparity between the two eyes of the same patient • ERG—rods and cones response impaired • fibrotic scar in 4/34 eyes • VF—bilateral paracentral scotomata, progressive with time in area and density • staging
Simon Dulz et al., 2021 [22]	• retrospective case series • cumulative long-chain 3-hydroxy fatty acids (3-OHFAs) assessment • follow-up: median 6.2 years (range 7.9–23.9 years)	6 patients	• patients diagnosed with LCHADD at the University Children’s Hospital of Hamburg-Eppendorf	• chorioretinopathy present in all cases • patients diagnosed by newborn screening revealed milder retinal involvement and retained visual function • impaired BCVA in 6/12 eyes • fibrotic scar in 2/6 patients (no data regarding whether in one or both eyes) • no obvious correlation between 3-OHFAs levels, the number of metabolic crisis, and the stage of chorioretinopathy and its progression • staphyloma in 4/12 eyes • staging
Kristina Rücklová et al., 2021 [18]	• aim: to analyze the impact of a newborn screening and early initiated nutritional management on clinical outcome in patients with LCHADD/TFPD	28 patients with LCHADD and TFPD	• almost all patients with LCHADD/MTPD born between 1984 and 2020 in Czech Republic	• severity of retinopathy did not differ between patients diagnosed with newborn screening and after presentation with clinical symptoms
Melanie B. Gillingham et al., 2024 [6]	• prospective cohort study on natural history study of LCHADD and TFPD retinopathy • aim: to evaluate the relationship of visual outcomes between patients managed after diagnosis due to symptomatic presentation and those diagnosed and treated following newborn screening or based on family history	37 LCHADD patients + 3 TFPD patients	• all patients diagnosed with LCHADD and TFPD at two clinical sites, OHSU and UPMC, from July 2020 to August 2022 (only patients ≥2 years old included)	• chorioretinopathy more severe among patients diagnosed as symptomatic than those identified by newborn screening or family history (regardless of the phenotype) • ERG: dark-adapted amplitudes were lower in older participants and were associated with genotype, with reduced amplitudes observed in individuals carrying one or two copies of c.1528G>C. Dark-adapted rod response times were prolonged in older participants and in those diagnosed based on symptomatic presentation. Photopic ERG amplitudes were diminished in participants who presented symptomatically compared with those diagnosed through newborn screening or family history. Photopic response times were significantly longer in older participants and in those diagnosed symptomatically • contrast sensitivity: scores were lower in older participants, associated with genotype, and reduced among those who presented symptomatically compared with participants diagnosed by newborn screening or family history • visual fields: changes were more severe in older patients and in those who presented symptomatically
Nida Wongchaisuwat et al., 2024 [23]	• prospective cohort study on natural history study of LCHADD (37 patients) and TFPD (3 patients) • aim: to develop an updated staging system for LCHADD chorioretinopathy	37 LCHADD patients + 3 TFPD patients	• all patients diagnosed with LCHADD and TFPD at two clinical sites, OHSU and UPMC, between July 2020 and August 2022 (only patients ≥2 years old included)	• 60/80 eyes with chorioretinopathy • 37/80 eyes BCVA impaired • 12/80 eyes presented with ERM • 9/80 eyes developed CNV • 21/80 eyes developed CME • Staphyloma in all eyes at stage 4
Magdalena Hubert et al., 2025 [24]	• retrospective case series • duration 9–16 years	8 patients	• all patients referred to Pomeranian Hospital with LCHADD	• chorioretinopathy present in all patients • myopia in 16/16 eyes • impaired BCVA in 8/19 eyes • CNV in 3/16 eyes: 1 active, 2 fibrotic scar • staphyloma in 7/16 eyes • VF—bilateral paracentral scotomata—with time enlarged and dense • active CNV manifested with retinal hemorrhage

ERG—electroretinography, LCHADD—long-chain 3-hydroxyacyl-CoA dehydrogenase deficiency, BCVA—best corrected visual acuity, WHO—World Health Organization, TFPD—trifunction protein deficiency, OCT—optical coherence tomography, VF—visual field, 3-OHFA—3-Hydroxy fatty acids, ERM—epiretinal membrane, CNV—choroidal neovascularization, CME—cystoid macular edema.

**Table 2:** Studies regarding macular neovascularization in LCHADD patients published in recent 20 years.

Study	Study Design	No of Patients	Population	Results
Nida Wongchaisuwat et al., 2023 [25]	• prospective cohort study—natural history study of LCHADD and TFPD • patients were identified as suspected of CNV when there was evidence of subretinal hemorrhage, fluid, scar, or a history of anti-VEGF therapy; OCT and OCTA examination performed to confirm the diagnosis	• 33 patients with LCHADD or TFPD	• all patients diagnosed with LCHADD and TFPD at two clinical sites, OHSU and UPMC, between July 2020 and August 2022, age: 2–60 years old that managed to undergo the OCT and OCTA examination	• the prevalence of CNV in at least one eye—21% • active CNV or scar in 27% eyes • patients with CNV in at least one eye were older than those with no evidence of CNV • 8/9 CNV were type 2, 1/9 CNV was type 1 • CNV appears usually at stage 2 • CNV is typically inactive, small, and focal • CNV can spontaneously regress or require only few anti-VEGF injections for resolution • onset from the age of 6
Magdalena Hubert et al., 2025 [26]	• retrospective study	• 8 patients	• all patients referred to Pomeranian Hospital with LCHADD	• CNV in at least one eye in 25% • CNV or scar in 19% eyes • onset from the age of 9 • treatment with intravitreal injections of ranibizumab led to complete resolution and recovery of full BCVA in one eye • inactive CNV/scar leads to deterioration of vision • CNV was type 2 in all cases

LCHADD—long-chain 3-hydroxyacyl-CoA dehydrogenase deficiency, TFPD—trifunction protein deficiency, VEGF—vascular endothelial growth factor, OCT—optical coherence tomography, OCTA—OCT angiography, CNV—choroidal neovascularization, BCVA—best corrected visual acuity, OHSU—Oregon Health & Science University, Portland, Oregon, USA, UPMC—the University of Pittsburgh Medical Center, Pittsburgh, Pennsylvania, USA.

**Table 3:** Studies on effects of dietary therapy in LCHADD patients published in recent 20 years.

Study	Study Design	No of Patients	Results
Melanie B. Gillingham et al., 2005 [16]	• prospective cohort study/open-label trial • aim: to determine whether DHA supplementation (65 mg/day for subject <20 kg; 130 mg/day for subject >20 kg) prevents the progression of the chorioretinopathy in LCHAD	• 11 LCHADD patients including 3 TFPD patients	• Levels of long-chain 3-hydroxyacylcarnitines negatively correlated with maximum ERG amplitude. • Neither plasma DHA concentrations nor age were independent predictors of any ERG outcomes. • VEP amplitudes increased with duration of DHA supplementation, regardless of baseline DHA status (normal or low). • No differences in VEP results observed between participants with low cumulative hydroxyacylcarnitine levels and those with high cumulative levels. • A trend toward a positive correlation observed between VEP measures and plasma DHA concentrations. • Optimal dietary therapy—indicated by low plasma 3-hydroxyacylcarnitine and high plasma DHA concentrations—associated with preservation of retinal function and BCVA.

DHA—docosahexaenoic acid, LCHAD—long-chain 3-Hydroxyacyl-CoA dehydrogenase, LCHADD—LCHAD deficiency, TFPD—trifunction protein deficiency, ERG—electroretinography, VEP—visual evoked potentials, BCVA—best corrected visual acuity.

## Data Availability

No new data were created or analyzed in this study. Data sharing is not applicable to this article.
